# Moving to a Highly Walkable Neighborhood and Incidence of Hypertension: A Propensity-Score Matched Cohort Study

**DOI:** 10.1289/ehp.1510425

**Published:** 2015-11-08

**Authors:** Maria Chiu, Mohammad-Reza Rezai, Laura C. Maclagan, Peter C. Austin, Baiju R. Shah, Donald A. Redelmeier, Jack V. Tu

**Affiliations:** 1 Institute for Clinical Evaluative Sciences, Toronto, Ontario, Canada; 2 Department of Medicine, University of Toronto, Toronto, Ontario, Canada; 3 Department of Medicine,; 4 Sunnybrook Research Institute, and; 5 Schulich Heart Centre, Sunnybrook Health Sciences Centre, Toronto, Ontario, Canada

## Abstract

**Background:**

The impact of moving to a neighborhood more conducive to utilitarian walking on the risk of incident hypertension is uncertain.

**Objective:**

Our study aimed to examine the effect of moving to a highly walkable neighborhood on the risk of incident hypertension.

**Methods:**

A population-based propensity-score matched cohort study design was used based on the Ontario population from the Canadian Community Health Survey (2001–2010). Participants were adults ≥ 20 years of age who moved from a low-walkability neighborhood (defined as any neighborhood with a Walk Score < 90) to either a high- (Walk Score ≥ 90) or another low-walkability neighborhood. The incidence of hypertension was assessed by linking the cohort to administrative health databases using a validated algorithm. Propensity-score matched Cox proportional hazard models were used. Annual health examination was used as a control event.

**Results:**

Among the 1,057 propensity-score matched pairs there was a significantly lower risk of incident hypertension in the low to high vs. the low to low-walkability groups [hazard ratio = 0.46; 95% CI, 0.26, 0.81, p < 0.01]. The crude hypertension incidence rates were 18.0 per 1,000 person-years (95% CI: 11.6, 24.8) among the low- to low-walkability movers compared with 8.6 per 1,000 person-years (95% CI: 5.3, 12.7) among the low- to high-walkability movers (p < 0.001). There were no significant differences in the hazard of annual health examination between the two mover groups.

**Conclusions:**

Moving to a highly walkable neighborhood was associated with a significantly lower risk of incident hypertension. Future research should assess whether specific attributes of walkable neighborhoods (e.g., amenities, density, land-use mix) may be driving this relationship.

**Citation:**

Chiu M, Rezai MR, Maclagan LC, Austin PC, Shah BR, Redelmeier DA, Tu JV. 2016. Moving to a highly walkable neighborhood and incidence of hypertension: a propensity-score matched cohort study. Environ Health Perspect 124:754–760; http://dx.doi.org/10.1289/ehp.1510425

## Introduction

There is growing interest in the impact of the built environment on the promotion of physical activity (McCormack and Shiell 2011; Witten et al. 2012) and the prevention of cardiovascular diseases (Ludwig et al. 2011; Sallis et al. 2012; Kumanyika et al. 2008). In particular, living in walkable neighborhoods (i.e., neighborhoods with shorter, more connected streets and with greater access to a variety of shops and other amenities within walking distance) has been associated with increased walking and decreased prevalence of obesity and other cardiovascular risk factors, including hypertension (Berry et al. 2010; Booth et al. 2013; Giles-Corti et al. 2008; Hirsch et al. 2013, 2014a, 2014b, 2014c; Mujahid et al. 2008; Müller-Riemenschneider et al. 2013). A recent analysis by our group using a similar representative sample of the Ontario population from Statistics Canada’s Canadian Community Health Survey found that living in higher Walk Score areas was significantly associated with more utilitarian walking and a decreased prevalence of obesity (Chiu et al. 2015). A major limitation of past work on neighborhood walkability and health outcomes, such as hypertension, has been the reliance on cross-sectional data (Casagrande et al. 2011; Müller-Riemenschneider et al. 2013). The use of these data raises methodological concerns regarding the potential for reverse causation—the outcome potentially preceding or causing the exposure instead of the other way around. Moreover, earlier studies have not been able to adequately adjust for important individual characteristics [e.g., income, education, marital status, body mass index (BMI)] that differ between people who live in low- and those in high-walkability neighborhoods and might influence their risk of hypertension independent of physical activity.

We conducted a population-based cohort study using propensity-score matching methods to examine the risk of incident hypertension among individuals who moved from a low- to a high-walkability neighborhood compared with individuals who moved from a low- to another low-walkability neighborhood.

## Methods

### Walk Score

Several walkability indices have been created for individual study settings (Booth et al. 2013; Frank et al. 2010; Toronto Public Health 2012); however, Walk Score’s Street Smart Walk Score (henceforth called Walk Score) is currently the only walkability index that is publicly available for all postal codes and ZIP codes in Canada, the United States, and Australia (http://www.walkscore.com). The Walk Score has been shown to be a valid measure for estimating neighborhood walkability in multiple geographic locations and at multiple spatial scales in the United States as measured based on significant moderate Spearman correlations with geographic information system–derived walkability indicators (Duncan et al. 2011). The Walk Score is based on walking distances from a given location to a diverse set of nearby amenities, including grocery stores, restaurants, shopping, coffee shops, banks, parks, schools, book stores and libraries, and entertainment. The points for each type of amenity are added and then normalized to yield a score from 0 to 100 with penalties of up to 5% applied for areas with lower street connectivity (Walk Score 2014).

### Data Sources and Study Cohort

The study was conducted at the Institute for Clinical Evaluative Sciences (ICES), a repository of linked administrative health databases, including individual-level data for hospital discharges and Ontario Health Insurance Plan (OHIP) physician and laboratory claims and the Canadian Institute for Health Information (CIHI) Discharge Abstract Database for hospital admissions for people in Ontario, Canada (unpublished data).

The study population consisted of Ontario participants of Statistics Canada’s Canadian Community Health Surveys (CCHS) (2001–2010) (Thomas and Wannell 2009). These surveys used a complex sampling strategy to collect sociodemographic and health information from a representative sample of Canadians ≥ 12 years of age living in private dwellings (including apartments). The surveys excluded institutionalized individuals, individuals living on Aboriginal reserves, full-time members of the Canadian forces, and residents of certain remote regions. The individual response rates in the different CCHS surveys ranged from 75.1% to 94.4%. More details about these surveys are found elsewhere (Desmeules 2004).

The outcome—incident hypertension—was derived through linkage of the survey data to the population-based Ontario Hypertension Database, which uses a validated algorithm of one CIHI hospital admission with a hypertension diagnosis or one OHIP claim with a hypertension diagnosis followed within 2 years by another OHIP claim or one CIHI hospital admission (specificity, 95%; sensitivity, 72% validated using primary care charts) (Tu et al. 2007, 2008).

**Figure 1:**
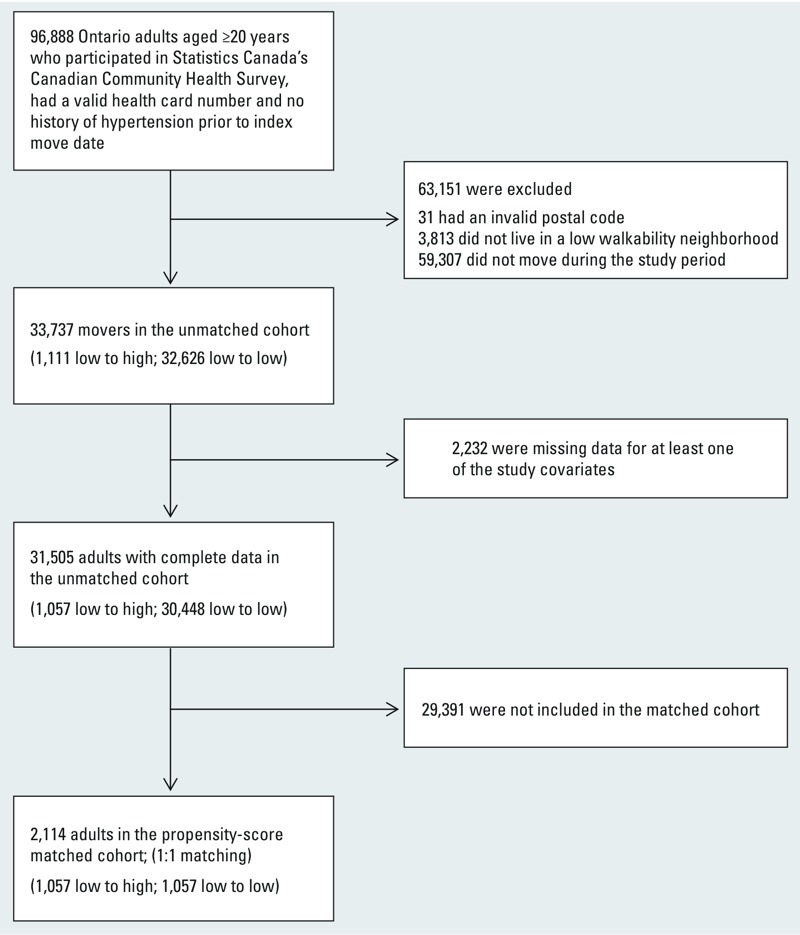
Study flow diagram. Low- and high-walkability areas were defined as Walk Score of < 90 and ≥ 90, respectively.

Figure 1 illustrates the creation of the study cohort. The cohort was restricted to CCHS respondents ≥ 20 years of age at their survey date who had a valid Ontario health card number and who did not have previous hypertension (as ascertained by self-report or through linkage to the Ontario Hypertension Database). For each study participant, we ascertained longitudinal annual postal codes of residence starting from the year of interview by linking our CCHS study population data set to the Registered Person’s Database (RPDB) from the Ontario Ministry of Health and Long-term Care (MOHLTC) (Health Analytics Branch 2012). The RPDB includes postal codes for all Ontario residents and is updated on 1 July of each year via linkage to other administrative databases at the Institute for Clinical Evaluative Sciences. The annual postal codes were then linked to a file purchased from Walk Score® (which contained Walk Scores as of 2012) to assign a Walk Score for each annual postal code, the smallest unit for which geographical information was available from the CCHS survey. Postal codes are defined by Canada Post Corporation for the efficient sorting and delivery of mail and represent small geographical units which may be made up of a specific city block in urban areas (one side of a street between two intersecting streets) or a rural community in rural areas. The cohort was limited to individuals who at the time of the survey were living in a low-walkability neighborhood and who subsequently moved neighborhoods following the interview date. Individuals were classified as either moving from a low- (defined as Walk Score < 90) to a high-walkability (defined as Walk Score ≥ 90) postal code (i.e., low to high group) or from a low- to a different low-walkability postal code (i.e., low to low group). Included in our definition of low-walkability postal codes are those defined by Walk Score as “Car-Dependent” (0-49), “Somewhat Walkable” (50–69) and “Very Walkable” postal codes (70–89). Our high-walkability postal code corresponds to “Walker’s Paradise” areas as defined by Walk Score (http://www.walkscore.com). The date of first move was defined as the index date. Individuals were followed using administrative health databases from index date to the date of incident hypertension and were censored at date of death (from the RPDB), date of move outside of Ontario, end of study date (1 July 2012), or date when an individual in the low to low group had a subsequent move to a high-walkability (Walk Score ≥ 90) postal code or when an individual in the low to high group had a subsequent move back to a low-walkability (Walk Score < 90) postal code.

### Study Covariates

The following covariates based on self-reported data collected at the time of survey were used to calculate propensity scores: age (used to calculate age at index date); sex; education (< secondary school vs. ≥ secondary school); marital status (married/common law vs. single/widowed/divorced); immigrant status (immigrant vs. non-immigrant); race/ethnicity (i.e., white, Chinese, South Asian, black, other); current smoking; diabetes (physician diagnosed); BMI (from self-reported weight and height); psychosocial stress (i.e., feeling extremely/quite a bit vs. not at all/not very/a bit stressed in most days); inadequate leisure physical activity (i.e., participating in at most 15 min of daily physical activity); alcohol consumption [i.e., regular drinker (≥ once per month), occasional drinker (> once per year but < once per month), or never in the past 12 months)]; and inadequate fruit and vegetable consumption (i.e., eating fruits or vegetables less than three times per day). We also included index year, as well as Statistics Canada’s 2006 census-derived area-based income quintiles (household-size adjusted income averaged at the dissemination area level, which generally includes a population of 400–700 individuals) and urban (≥ 10,000 population) or rural (< 10,000 population) dwelling at index date in the propensity-score models. The covariates were chosen based on *a priori* hypotheses and walkability literature, as well as all available factors associated with walkability and/or hypertension based on previous studies. A separate multivariable Cox proportional-hazards model using the unmatched sample was also constructed including all of the covariates mentioned above.

### Control Event

We assessed annual health examination as the control event. Control or tracer events have been used in previous studies to detect possible biases by testing for the lack of association between the exposure and the control event when there is expected to be no association. (Hackam et al. 2006). Annual health examination, a general health assessment for patients with no apparent physical or mental illness, was chosen as the control event to examine possible differences in health care seeking behavior using an event unlikely to be related to hypertension or physical activity. We used the same propensity score–matched cohort as the main analysis and linked to the OHIP database to ascertain time to the first annual health examination during the follow up period.

### Statistical Analyses

SAS v. 9.3 (SAS Institute Inc.) and R v. 3.1.2 (R Core Team 2014) were used for statistical analysis. All tests were two-sided and *p* < 0.05 was considered statistically significant. Study data sets were linked using unique encoded identifiers and analyzed at the ICES. All estimates were weighted using Statistics Canada’s original survey weights to generate results that are representative of the overall Ontario population.

### Propensity Score–Matched Analysis

A propensity score for the probability of moving from a low- to a high-walkability postal code was estimated for each individual using a weighted logistic regression model including the 16 study covariates previously stated. We created a propensity score–matched cohort by attempting to match each participant in the low to high group to an individual in the low to low group. A nearest-neighbor-1:1-greedy matching algorithm was applied to match participants on the basis of the logit of their propensity score, with a caliper width equal to 0.2 times the standard deviation of the logit of the propensity score (Austin 2011; Austin and Small 2014). Balance of baseline covariates between the exposed and control groups in the matched sample was assessed using standardized differences, with standardized differences of < 0.1 for each covariate being used to indicate good balance (Austin 2009). We also assessed whether the groups were balanced on other health status and individual income variables that were not used to derive propensity scores, including self-reported health, mental health, and continuous individual-level income.

The effect of moving to a high-walkability neighborhood (compared with moving to a low-walkability neighborhood) was estimated using a Cox proportional hazards model that regressed the hazard of incident hypertension on the exposure group. To account for the paired nature of the matched sample, robust sandwich-type variance estimators were used to assess the statistical significance of the estimated hazard ratio (Austin 2013). Survival curves were produced using Kaplan–Meier methods. All analyses were weighted by the survey sample weights and appropriate propensity-score matching and bootstrap methods for complex survey design were applied (Austin and Small 2014; Zanutto 2006).

### Unmatched Sample and Sensitivity Analyses

To assess whether results were consistent using the entire sample of survey respondents, we also calculated adjusted Cox proportional hazard ratios and bootstrapped *p*-values for the incidence of hypertension among the larger unmatched sample (low to low *n* = 32,626; low to high *n* = 1,111). This was important in order to verify that results were consistent using traditional multivariable regression methods. Adjusted survival curves were produced using the corrected group prognosis method (Makuch 1982; SAS Institute Inc. 2014). For sensitivity analyses, we calculated Cox proportional hazard ratios for *a*) a sample limited to only those who lived in non-rural postal codes at the time of the survey and censoring occurring upon moving to a rural postal code (*n* = 26,048), and *b*) a sample where a cut point of Walk Score 70 was used instead of 90 (*n* = 26,563) to dichotomize high- and low-walkability neighborhoods.

### Ethics Committee Approval

Our study was approved by the Research Ethics Board at Sunnybrook Health Sciences Centre. Informed consent for the use of data for research purposes was obtained from all survey participants by Statistics Canada.

## Results

### Study Population

The unmatched sample included a total of 33,737 adults (before excluding participants with missing covariate data): 1,111 in the low to high group and 32,626 in the low to low group. The baseline characteristics of the unmatched sample are displayed in Table S1. In the unmatched sample, the movers in the low- to low-walkability group were on average older (39.9 years vs. 37.0 years) and less likely male (48.3% vs. 51.7%) than those in the low- to high-walkability group. Levels of less than secondary school education (11.3% vs. 5.5%), the prevalence of overweight/obesity (43.6% vs. 33.0%) and diabetes (1.9% vs. 0.6%), as well as leisure physical activity (inactive 51.1% vs. 46.5%) and alcohol consumption (regular drinker; 64.2% vs. 72.9%) were similar between the low- to low- and the low- to high-walkability groups, respectively.

### Propensity-Score Matched Analysis

After propensity-score matching, a total of 1,057 (95%) low to high movers were matched to 1,057 low to low movers. The matched sample was balanced, with standardized differences ≤ 0.01 for all comparisons (Table 1). The matched cohort was followed for up to 10 years, with a mean length of follow-up of 4.3 years (median, 4.0; range, 0.03–11.0) in the low to low group compared with 3.0 years (median, 2.0; range, 0.03–11.0) in the low to high group. Mean individual-level income was well balanced between the two study groups ($34,311 in the low to low group vs. $37,030 in the low to high group). The proportion of individuals in middle and high income area–based income quintiles was also well balanced (49.1% in the low to low group vs. 49.2% in the low to high group) (Table 1). The mean Walk Score before move in the low to low movers was 39.7 (median, 41; range, 0–89) compared to 50.2 (median, 55; range, 0–89) in the low to high movers. The mean post-move Walk score in the low to low movers was 40.0 (median, 41.0; range, 0–89) compared with 94.4 (median, 94; range, 90–100) in the low to high movers.

**Table 1:** Baseline characteristics of the propensity-score matched cohort.

Sociodemographic characteristics	Low to low walkability (*n *= 1,057)	Low to high walkability (*n *= 1,057)	Standardized difference
Age at index date (years) [mean (median)]	36.8 (33)	37.0 (34)	< 0.01
Age at index date (grouped) (years)
20–34	55.6	54.0	< 0.01
35–45	19.0	22.2	< 0.01
≥ 46	25.4	23.9	< 0.01
No. of years between interview and index dates [mean (median)]	2.9 (2)	3.2 (2)	0.01
Male sex (%)	49.3	52.7	< 0.01
Area-based income quintile at index date
1 (lowest)	27.3	26.4	< 0.01
2	23.6	24.4	< 0.01
3	15.2	14.3	< 0.01
4	13.7	13.6	< 0.01
5 (highest)	20.2	21.3	< 0.01
Individual-level income ($) [mean (median)]	34,311 (28,000)	37,030 (30,000)	< 0.01
Less than secondary school education	4.0	5.0	< 0.01
Married or common-law	29.5	32.4	< 0.01
Urban dwelling at index date	92.3	93.8	< 0.01
Immigrant	33.3	34.5	< 0.01
No. of years in Canada (among immigrants) [mean (median)]	16.1 (12)	15.8 (13)	< 0.01
Race/ethnicity
White	70.0	71.5	< 0.01
South Asian	1.6	2.9	< 0.01
Chinese	4.7	3.8	< 0.01
Black	7.7	5.6	< 0.01
Other	16.1	16.2	< 0.01
Current smoker	26.6	28.6	< 0.01
Prevalent diabetes	0.3	0.7	< 0.01
BMI (kg/m^2^) [mean (median)]	24.0 (23)	24.0 (24)	< 0.01
Overweight (BMI ≥ 25 kg/m^2^)	34.7	33.1	< 0.01
Obese (BMI ≥ 30 kg/m^2^)	7.7	7.1	< 0.01
Psychosocial stress	29.7	30.2	< 0.01
Leisure physical activity
Active	34.1	29.4	0.01
Moderate	21.1	24.7	< 0.01
Inactive	44.8	45.9	< 0.01
Leisure physical activity (≤ 15 min/day)^*a*^	60.4	62.0	< 0.01
Alcohol consumption^*b*^
Regular drinker	72.5	74.3	< 0.01
Occasional drinker	13.1	11.8	< 0.01
Nondrinker	14.4	13.9	< 0.01
Inadequate fruits and vegetables (< 3 times per day)	22.6	25.0	< 0.01
No. of times consumed fruits and vegetables per day [mean (median)]	4.9 (4)	5.0 (5)	< 0.01
Poor/fair self-rated overall health	6.4	6.9	< 0.01
Poor/fair self-rated mental health	4.3	4.5	< 0.01
Low- and high-walkability areas were defined as Walk Score of < 90 and ≥ 90, respectively. Data were derived from the Ontario components of Canadian Community Health Survey (2001–2010) linked to the Ontario Hypertension Database. Estimates are percentages or mean (median). All estimates were weighted by the survey sample weight. In all comparisons of characteristics, the groups were well balanced (standardized differences in the mean ≤ 0.01 for all comparisons). ^***a***^Leisure physical activity [average daily energy expenditure (active: ≥ 3.0 kcal/kg/day; moderately active: 1.5–2.9 kcal/kg/day; inactive: < 1.5 kcal/kg/day)] ^***b***^Alcohol consumption (regular drinker: at least once per month; occasional drinker: less than once per month; nondrinker: never in the past year from survey date).			

**Figure 2:**
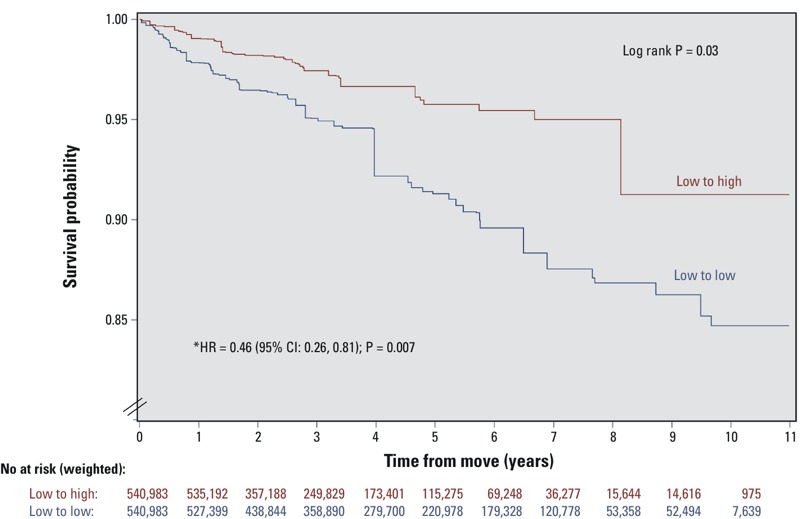
Event-free survival for incident hypertension in a propensity score–matched cohort of participants who moved from low- to high-walkability areas vs. from low- to low-walkability areas. Low- and high-walkability areas were defined as Walk Score of < 90 and ≥ 90, respectively. The *p*-value tests the difference between the Kaplan–Meier survival curves using the log-rank test. All estimates were weighted by the survey sample weights and bootstrap methods were applied. The hazard ratios, 95% confidence intervals, and *p*-values were derived from a Cox proportional hazards model performed on the propensity score–matched study sample of 1,057 pairs of participants balanced on age, sex, income, education, marital status, urban/rural residence, immigrant status, race/ethnicity, smoking, diabetes, BMI, stress, leisure physical activity, alcohol consumption, fruit and vegetable consumption, and index year.

There was a significantly lower risk of incident hypertension in the low to high versus low to low groups [hazard ratio (HR) = 0.46; 95% confidence interval (CI): 0.26, 0.81, *p* < 0.01]. The crude hypertension incidence rates were 18.0 per 1,000 person-years (95% CI: 11.6, 24.8) in the low to low movers compared with 8.6 per 1,000 person-years (95% CI: 5.3, 12.7) in the low to high movers (*p* < 0.001). Figure 2 displays the event-free Kaplan–Meier curves for the two study groups.

### Control Event

**Figure 3:**
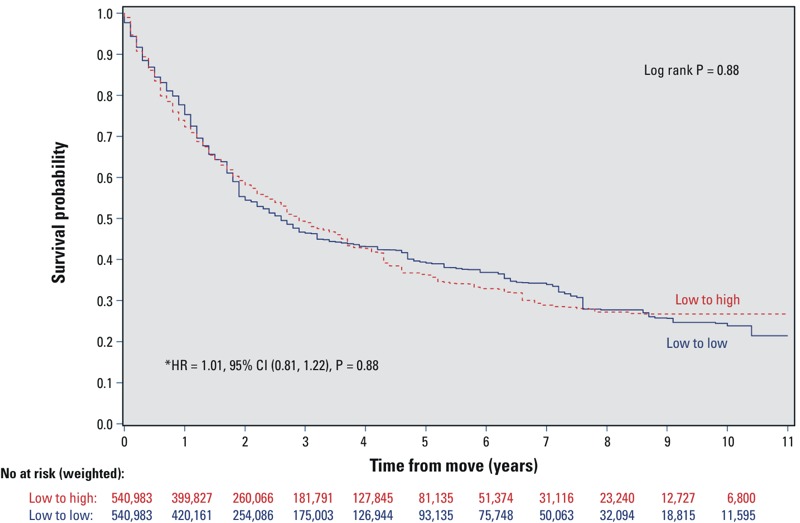
Kaplan–Meier survival curves for annual health examination in a propensity score–matched cohort of movers from low- to high-walkability areas vs. from low- to low-walkability areas. Low- and high-walkability areas were defined as Walk Score of < 90 and ≥ 90, respectively. Kaplan–Meier survival curves were weighted using survey weights. The *p*-values test the differences between the Kaplan–Meier survival curves using the log-rank test. The hazard ratios, 95% confidence intervals, and *p*-values were derived from Cox proportional hazards models performed on the propensity score–matched study sample, which was balanced on age, sex, income, education, marital status, urban residence, immigrant status, race/ethnicity, smoking, diabetes, BMI, stress, leisure physical activity, alcohol consumption, fruit and vegetable consumption, and index year.

There was no significant difference in the hazard of annual health examination (HR = 1.01; 95% CI: 0.85, 1.22, *p* = 0.88) between the two study groups. Figure 3 displays the Kaplan–Meier curves for this relationship.

### Unmatched Sample and Sensitivity Analyses

Similar results to the main analysis were obtained when hazard ratios were calculated for the unmatched sample adjusted for the same 16 covariates included in the propensity score matched analysis (HR = 0.57; 95% CI: 0.35, 0.85, *p* = 0.01) (see Table S2 and Figure S1), and for the sample that excluded rural dwellers (HR = 0.58; 95% CI: 0.36, 0.86, *p* = 0.02).

When the threshold for what constituted a “high” walkability area was lowered from Walk Score 90 to 70, as expected, we found an attenuated but still negative association of moving to a highly walkable neighborhood on hypertension incidence among the unmatched sample adjusted for the same 16 covariates (HR = 0.81; 95% CI: 0.66, 1.02, *p* = 0.06).

## Discussion

In this large population-based sample, we found that moving to an area with a very high Walk Score (indicating a neighborhood that is very conducive to utilitarian walking) was associated with a significantly lower risk of hypertension. People who moved from low- to high-walkability areas had a 54% lower risk of incident hypertension than their counterparts in the propensity score–matched low- to low-walkability group. There was no significant difference in the hazards of the control event—annual health examination—between the two study groups.

Our findings are consistent with earlier evidence that increased neighborhood walkability is associated with increased walking (Owen et al. 2010; Sundquist et al. 2011) and lower prevalence of obesity and hypertension (Berry et al. 2010; Mujahid et al. 2008; Müller-Riemenschneider et al. 2013). A recent study of adults in Australia followed participants moving to a new residential development over a 7-year period and found that neighborhood walkability, access to public transit stops, and having a variety of local destinations were predictors of whether participants walked for transportation in their neighborhood (Knuiman et al. 2014). A systematic review of experimental and observational studies examining the association between built environments and physical activity found that neighborhood amenities, street connectivity, and population density (similar attributes used to derive the Walk Score) were important determinants of physical activity, particularly transportation or utilitarian walking (McCormack and Shiell 2011). Moreover, other survey-based studies have reported that movers walked and biked more 1 year post-move if the neighborhood to which they moved included an increase in the mix of businesses within walking distance of their residence (Cao et al. 2007; Handy et al. 2006). Because of the impracticability and cost of a randomized control trial to answer our research question, we performed a propensity score–matched analysis of prospective data using a natural history experiment of people’s moving patterns. Our study findings are analogous and consistent in magnitude and direction to two randomized studies that have suggested that neighborhood environments can directly influence health and reduce risk of hypertension (He et al. 2000; Ludwig et al. 2011). One such study, phase one of the Trials of Hypertension Prevention, found a 77% reduction in the odds of hypertension among those receiving the lifestyle intervention, which included brisk walking (He et al. 2000). Similarly, the Moving to Opportunity project found that the opportunity to move from a neighborhood with a high level of poverty to one with a lower level of poverty was associated with a reduction in the prevalence of extreme obesity and diabetes, thus suggesting that neighborhood characteristics have the potential to improve cardiovascular health (Ludwig et al. 2011).

We recognize that there may be several other neighborhood characteristics associated with highly walkable areas that may have contributed to our findings. For example, walkable neighborhoods often have easier access to transit, and it has been shown that people who take transit generally are more likely to meet daily physical activity recommendations (Lachapelle and Frank 2009) and have a lower BMI (Flint et al. 2014; Duncan et al. 2014). We also acknowledge that there are some negative consequences to living in highly walkable areas; for example, these areas may have substantial variation in other characteristics of highly walkable areas, such as higher levels of noise and air pollution, which have been shown to negatively impact health (Frank and Engelke 2005; Moudon 2009). However, some studies have found that higher walkability areas are associated with lower levels of air pollution (Frank et al. 2006). Future studies should also consider other characteristics that may be associated with walkability and both physical and mental health, including the food environment (Rundle et al. 2009) and pollutants (Marshall et al. 2009).

This study has several strengths. First, to our knowledge, our study represents the first cohort study to investigate the association between neighborhood walkability and risk of incident hypertension in a population-based sample, thus ensuring temporality between exposure and outcome. Second, we were able to adjust for 16 important study covariates, including many of the known risk factors for hypertension. Third, to optimize the comparability of the two study groups, we designed our study population to include only those who moved during the study period. All study participants also had to have lived in a low-walkability area at baseline, thus making the two study groups more similar than if a comparison was done for movers from low- to high- versus high- to low-walkability areas. Our estimates were weighted using survey weights, which allowed estimates to be generalizable to the overall Ontario population.

This study has limitations worth noting. First, we did not have serially measured blood pressure data. We also did not have detailed dietary data (e.g., salt intake) as well as more detailed measures of physical activity. Second, people moving to high-walkability neighborhoods may be healthier and/or demonstrate more health-seeking behavior. In this study, however, the propensity score–matching method ensured that the two mover groups were balanced on several lifestyle and health status covariates. In addition, we found no differences between the two study groups for annual health examination, thus suggesting that the groups likely did not differ in their health-seeking behavior. Third, we acknowledge that based on our dichotomization of Walk Score and classification of high-walkability areas (Walk Score ≥ 90), there may have been individuals living in walkable neighborhoods that were classified as low walkability. There also may have been variations in the change in Walk Score of individuals following their move both within and between groups. Fourth, we did not have information about other built environment attributes, such as street aesthetics and safety, which may influence physical activity and in turn may influence the risk of hypertension. Fifth, there were differences in the median years of follow-up between the low to low and low to high movers groups that should be acknowledged. Finally, a key assumption of propensity score modeling is that most observed confounding is accounted for; however, there remains the possibility that there may be residual confounding among unobserved covariates, such as other geographic factors or changes in sociodemographic characteristics, that could contribute to the results of the study. Future studies could focus on the effects of subcomponents of the Walk Score (e.g., amenities, density, land-use mix) and whether the relationship between moving to areas of higher walkability and a decreased risk of hypertension might differ across age, sex, and socioeconomic groups.

## Conclusions

In this large cohort study, moving to a highly walkable neighborhood was associated with a significantly lower risk of incident hypertension, a leading global burden of disease risk factor (Lim et al. 2012). Despite continued public health efforts to encourage people to participate in physical activity, only a small proportion of adults meet the minimum recommended physical activity levels to achieve health benefits (Colley et al. 2011). Thus, it becomes pertinent to emphasize that features of the built environment have the potential to encourage active living and improve population health, sentiments that are echoed by the American Heart Association (Bambs et al. 2011; Pearson et al. 2013), the World Health Organization’s European Healthy Cities Network (Edwards and Tsouros 2008), and the U.S. Surgeon General (2010). Our findings suggest that neighborhood walkability can positively affect health and may help raise awareness among the public of the importance of neighborhood environments.

## Supplemental Material

(314 KB) PDF
