# Supplementary material for: Moving to a Highly Walkable Neighborhood and Incidence of Hypertension: A Propensity-Score Matched Cohort Study
Source: Environ Health Perspect. 2015 Nov 8;124(6):754–60. doi: 10.1289/ehp.1510425 (PMC4892930; doi:10.1289/ehp.1510425)
Supplement: (314 KB) PDF [file ehp.1510425.s001.acco.pdf]

Environ Health Perspect
DOI: 10.1289/ehp.1510425

Note to readers with disabilities: *EHP* strives to ensure that all journal content is accessible to all readers. However, some figures and Supplemental Material published in *EHP* articles may not conform to 508 standards due to the complexity of the information being presented. If you need assistance accessing journal content, please contact ehp508@niehs.nih.gov. Our staff will work with you to assess and meet your accessibility needs within 3 working days.

Supplemental Material

Moving to a Highly Walkable Neighborhood and Incidence of Hypertension: A Propensity-Score Matched Cohort Study

Maria Chiu, Mohammad-Reza Rezai, Laura C. Maclagan, Peter C. Austin, Baiju R. Shah, Donald A. Redelmeier, and Jack V. Tu

Table of Contents

Table S1. Baseline characteristics of Ontario residents who moved from low to high walkability areas vs. low to low walkability areas, before Propensity-Score Matching.

Table S2. Cox-Proportional hazard ratios (HR) for the risk of incident hypertension in the unmatched sample of movers.

Figure S1. Event-free Adjusted Survival Curves for the Unmatched Sample of Participants who Moved from Low to High or Low to Low Walkability Areas. Low and high walkability areas were defined as Walk Score of <90 and ≥90, respectively. Survival curves were produced using the corrected group prognosis method (Makuch 1982). The model was adjusted for age, sex, income, education, marital status, immigrant status, urban/rural dwelling, race/ethnicity, current smoking, prevalent diabetes, inadequate leisure physical activity, inadequate fruit and vegetable consumption (<3 times per day), body-mass index, alcohol consumption, psychosocial stress, and index year. *Hazard ratios and p-values were produced using a weighted Cox proportional hazards model adjusted for the same covariates.

Table S1. Baseline characteristics of Ontario residents who moved from low to high walkability areas vs. low to low walkability areas, before Propensity-Score Matching.*

Sociodemographic Characteristics	Low to low walkability (n=32626)	Low to high walkability (n=1111)	P Value
Age at index date, years			
Mean (Median)	39.9 (38)	37.0 (34)	<0.001
Age at index date (grouped), years			
20 to 34	41.2	54.4	<0.001
35 to 45	29.3	21.9	<0.001
46+	29.4	23.8	0.004
No. of years between interview and index dates			
Mean (Median)	3.0 (2)	3.1 (2)	0.23
Male sex, %	48.3	51.7	0.14
Area-based income quintile at index date			
1 (lowest)	19.5	27.3	<0.001
2	19.6	23.7	0.06
3	20.4	14.6	<0.001
4	22.1	13.9	<0.001
5 (highest)	18.4	20.5	0.28
Individual-level income, $			
Mean (Median)	37,095 (30,000)	36,562 (28,000)	0.79
Less than secondary school education	11.3	5.5	<0.001
Married or common-law	59.7	33.0	<0.001
Urban dwelling at index date	90.4	93.6	0.001
Immigrant	30.5	34.8	0.08
Number of years in Canada (among immigrants)			
Mean, (Median)	16.3 (13)	15.6 (13)	0.46
Race/Ethnicity			
White	75.2	72.3	0.20
South Asian	6.4	3.2	<0.001
Chinese	3.6	3.6	0.99
Black	3.2	5.4	0.07
Other	11.6	15.6	0.05
Current smoker	29.6	28.7	0.68
Prevalent diabetes	1.9	0.6	<0.001
BMI (kg/m^2)			
Mean (Median)	25.1 (24)	24.0 (24)	<0.001
Overweight (BMI ≥ 25 kg/m^2)	43.6	33.0	<0.001
Obese (BMI ≥ 30 kg/m^2)	12.2	7.2	<0.001

Psychosocial stress	26.2	29.6	0.11
Leisure physical activity			
Active	24.7	29.4	0.04
Moderate	24.2	24.1	0.97
Inactive	51.1	46.5	0.10
Leisure physical activity (<15 mins/day)	65.1	61.8	0.14
Alcohol consumption			
Regular drinker	64.2	72.9	<0.001
Occasional drinker	17.4	11.5	<0.001
Non-drinker	18.4	15.6	0.11
Inadequate fruits and vegetables (<3 times per day)	26.2	25.3	0.65
No. of times consumed fruits and vegetables per day			
Mean (Median)	4.7 (4)	5.0 (5)	0.03
Fair/poor self-rated overall health	7.3	7.1	0.88
Fair/poor self-rated mental health	5.1	4.8	0.71

*Low and high walkability areas were defined as Walk Score of <90 and ≥90, respectively (Walk Score Methodology www.walkscore.com). Data were derived from the Ontario components of the Canadian Community Health Survey (2001-2010) linked to the Ontario Hypertension Database. Estimates are percentages unless otherwise specified. All estimates were weighted by the survey sample weight and bootstrap p-values were obtained. Definitions: leisure physical activity average daily energy expenditure (active: ≥3.0 kcal/kg/day, moderately active: 1.5-2.9 kcal/kg/day; inactive: <1.5 kcal/kg/day), alcohol consumption (regular drinker: at least once per month; occasional drinker: less than once per month; non-drinker: never in the past year from survey date).

Table S2. Cox-Proportional hazard ratios (HR) for the risk of incident hypertension in the unmatched sample of movers.*

Model	N			HR	95% Confidence Interval	P value
	Total	Low to low walkability	Low to high walkability			
Unadjusted	33737	32626	1111	0.51	(0.34, 0.74)	0.001
Adjusted for age and sex	33737	32626	1111	0.55	(0.37, 0.80)	0.004
+ income, education	33433	32327	1106	0.56	(0.37, 0.81)	0.004
+immigrant status, race/ethnicity, urban, married or common-law	33376	32270	1106	0.56	(0.37, 0.81)	0.006
+ leisure physical activity, BMI, smoking, diabetes, index year	31122	30561	1061	0.59	(0.36, 0.86)	0.02
+fruit/vegetable consumption, stress, alcohol consumption	31505	30448	1057	0.57	(0.35, 0.85)	0.01

*Low and high walkability areas were defined as Walk Score of <90 and ≥90, respectively. Data were derived from the Ontario components of Statistics Canada's Canadian Community Health Survey (2001-2010) linked to the Ontario Hypertension Database to ascertain incident hypertension. All estimates were weighted by the survey sample weight and bootstrap methods were used for variance estimation. Study covariates in the fully adjusted model included: age at index date, sex, area-based income quintiles from census data at index date, less than high school education, immigrant status, ethnicity (White, South Asian, Chinese, Black, other), urban or rural dwelling at index date, marital status, current smoking, body-mass index, prevalent diabetes, inadequate leisure physical activity, index year (ranging from 2001 to 2010), inadequate fruit and vegetable consumption (i.e. eating fruits or vegetables less than 3 times a day), psychosocial stress (i.e. feeling “extremely” or “quite a bit” vs. “not at all”, “not very” or “a bit” stressed in most days), and alcohol consumption (regular drinker, occasional drinker, never drank in past 12 months).

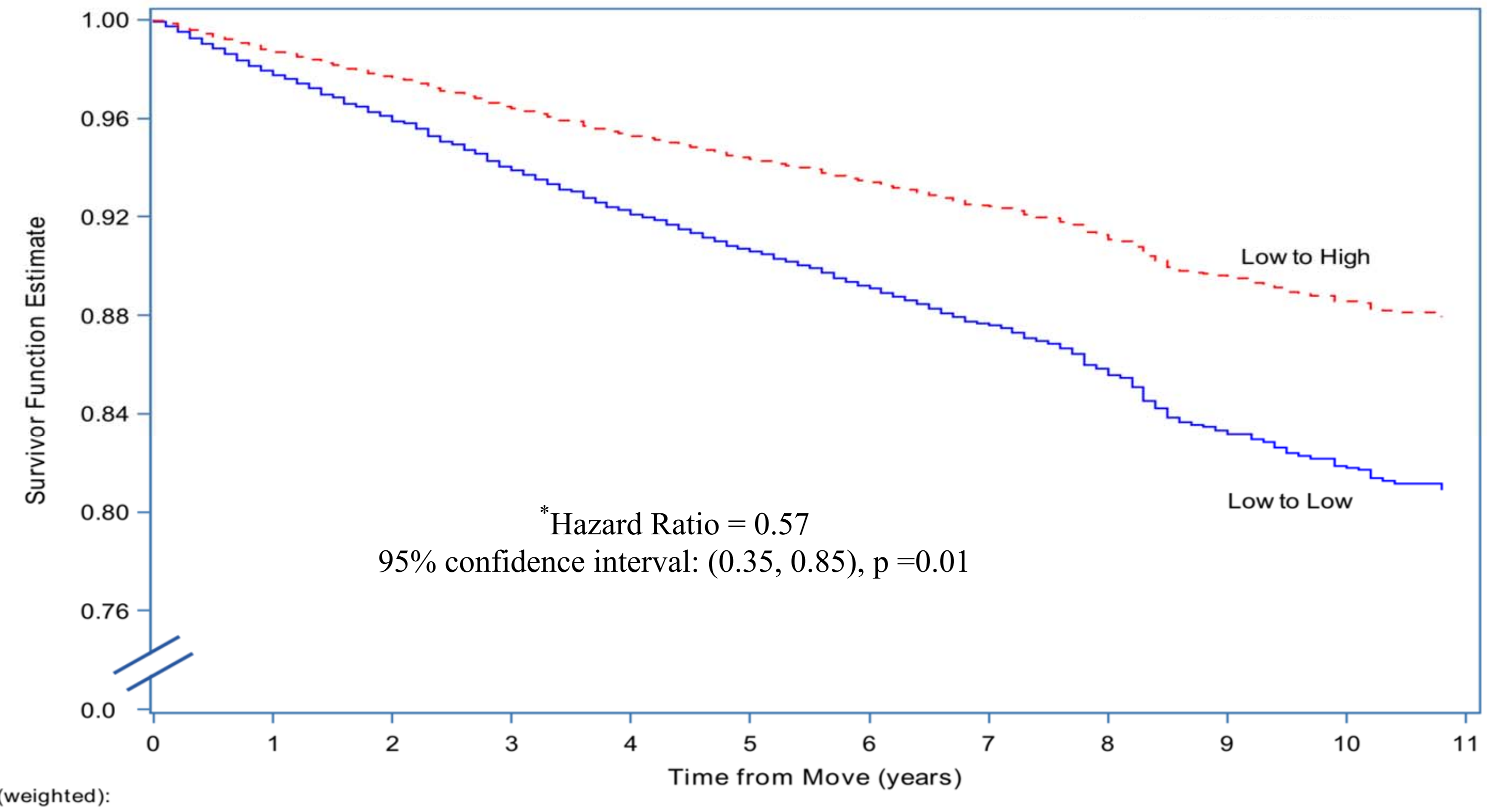

No at Risk (weighted):

Low to High:	541,777	535,994	356,844	249,586	173,230	115,155	69,179	36,240	15,631	14,604	974
Low to Low:	10,467,087	10,230,813	8,543,357	7,106,908	5,876,858	4,749,909	3,727,952	2,513,545	983,608	957,775	107,284

Figure S1. Event-free Adjusted Survival Curves for the Unmatched Sample of Participants who Moved from Low to High or Low to Low Walkability Areas.

Low and high walkability areas were defined as Walk Score of <90 and ≥90, respectively. Survival curves were produced using the corrected group prognosis method (Makuch 1982). The model was adjusted for age, sex, income, education, marital status, immigrant status, urban/rural dwelling, race/ethnicity, current smoking, prevalent diabetes, inadequate leisure physical activity, inadequate fruit and vegetable consumption (<3 times per day), body-mass index, alcohol consumption, psychosocial stress, and index year. *Hazard ratios and p-values were produced using a weighted Cox proportional hazards model adjusted for the same covariates.

References

Makuch RW. Adjusted survival curve estimation using covariates. 1982. J Chron Dis 35:437-443.

Walk Score Methodology. Available: http://www.walkscore.com/methodology.shtml [accessed 1 May 2015].
